# T700 Carbon Fiber/Epoxy Resin Composite Material Hygrothermal Aging Model

**DOI:** 10.3390/ma18020369

**Published:** 2025-01-15

**Authors:** Jinjie Lu, Chuanxiang Zheng, Liang Wang, Yuchen Dai, Zhenyu Wang, Zhaobo Song

**Affiliations:** 1 Institute of Process Equipment, College of Energy Engineering, Zhejiang University, Hangzhou 310007, China; 2 Department of Engineering Mechanics, School of Naval Architecture, Ocean and Civil Engineering, Shanghai Jiao Tong University, Shanghai 200240, China

**Keywords:** moisture absorption and diffusion, composite materials, hygrothermal aging, marine environmental, glass transition temperature

## Abstract

The hygrothermal aging model, based on Fick’s second law of diffusion, characterizes the degradation of engineering constants in T700 carbon fiber/epoxy resin composites. It focuses on changes in the tensile modulus, shear modulus, and transverse Poisson’s ratio due to moisture absorption and temperature variations. The model validates through mass change observations before and after seawater immersion, along with surface morphology assessments and tensile experiments. The results reveal that the saturated moisture absorption rate for single-layer laminates in seawater immersion is 0.35%. Short-term seawater immersion at room temperature (≤60 days) does not induce cracks or defects (≥10 μm) on the composite’s surface. The composite’s modulus decreases as moisture absorption increases, with the longitudinal tensile modulus dropping by an order of 10^−5^%, while the other engineering constants decrease by an order of 10^−3^%. The modulus also decreases with rising temperature; the closer the temperature is to the matrix’s glass transition, the faster the modulus declines, with the longitudinal tensile modulus decreasing by 0.84%, and the other engineering constants decreasing by 100%. This research provides valuable insights for the engineering applications of composite materials in marine environments.

## 1. Introduction

Fiber-reinforced polymer/plastic (FRP) is a type of composite material, and its exceptional mechanical properties are increasingly being harnessed, surpassing the capabilities of the individual component materials [1,2,3,4]. Aceti et al. reported instances where hygrothermal aging led to composite material failures in several aircraft models, including the Concorde, F-18, Boeing 767, and Airbus A330-2000 [5]. According to de Zeeuw et al., the effects of hygrothermal aging on composites used in tidal generators—where components are fully submerged in seawater—are notably more severe than those observed in wind turbine blades and aerospace applications [6]. As a result, when FRPs are deployed in real-world settings, it is crucial to evaluate their environmental adaptability [7,8]. In such applications, common service environments encompass moisture, immersion, high temperatures, hygrothermal conditions, corrosive media, and exposure to ultraviolet radiation, frequently coupled with hygrothermal cyclic conditions [9,10]. These environments can have irreversible effects on multiple properties of FRP [11], including the degradation of its mechanical properties [5]. Under marine conditions, the compressive strength of multidirectional laminate and orthogonal laminate decreases by approximately 50%, while the compressive strength of unidirectional laminate decreases by about 28% [12]. Therefore, further investigation into the effects of seawater immersion on FRP is of great significance for the safe application of FRP structural components.

Furthermore, extensive research has been conducted by scholars on the degradation mechanism of the mechanical performance of FRP under environmental conditions [13,14,15]. This mechanism can be summarized into three aspects, ranked in descending order of probability of occurrence: matrix cracking, interface debonding, and fiber fracture [7,16]. The degradation mechanism of FRP in a moist environment involves the penetration of water molecules into the matrix through diffusion and capillary action, leading to the hydrolysis and plasticization of the resin matrix. The hydrolysis process causes the resin matrix to soften and crack. Additionally, molecular chain breakage occurs during the plasticization process, resulting in a decrease in the matrix performance. Another portion of water molecules penetrates through the matrix to reach the matrix/fiber interface, causing the interface to swell, crack, and debond, leading to the mechanical degradation of the FRP [17]. In the case of reinforcing fibers, particularly carbon fibers, the probability of fracture in a humid and hot environment is extremely low and is generally disregarded. The bonding performance of the fiber–resin matrix interface of FRP is closely linked to the overall performance of the composite material. Therefore, interface debonding becomes one of the main forms of composite material failure in a humid and hot environment [18,19]. Understanding these failure mechanisms can aid in exploring the hygrothermal aging model. For instance, Nandagopal et al. established the relationship between the strength and failure strain of unidirectional laminates and the moisture absorption rate. The results indicate an inverse relationship between flexural, tensile, and compressive strength and the moisture absorption rate, as well as a similar inverse relationship between tensile and flexural failure strain and the moisture absorption rate [20].

With the continuous advancement of composite material manufacturing processes, an increasing number of domestically produced high-performance composite materials have been successfully applied in engineering, including the commonly used T700 carbon fiber/epoxy resin composite materials in the civil field. The research on the degradation of their mechanical properties in humid and hot environments still needs further enrichment. Therefore, this study explores the durability of domestically produced T700 carbon fiber/epoxy resin composite materials in a seawater corrosion environment, and demonstrates the applicability of introducing Fick’s second law and the material performance hygrothermal aging model to describe the variation of engineering constants of composite materials. The experimental results confirm the model’s relevance and guidance in exploring the durability issues of composite materials in environmental conditions.

## 2. Establishing Model for the Mechanical Performance Hygrothermal Aging

### 2.1. Moisture Absorption and Diffusion Characteristics

The moisture absorption process of fiber-reinforced composite materials is the diffusion of water molecules within the material, following Fick’s law. Therefore, a theoretical model can be used to describe the moisture absorption process and absorption rate of composite materials. Fick’s law comprises the first law and the second law, and this model is also widely applied in the moisture absorption model of polymer-based composite materials [21,22]. Fick’s second law describes the change in substance concentration over time in the mass transfer process, as shown in Equation (1), where the rate of change of water molecule concentration at a specific position inside the material is proportional to the product of the diffusion coefficient and the second derivative of the concentration gradient with respect to time.

(1) $$\frac{\partial c}{\partial t}=D_{z}\frac{\partial^{2}c}{\partial z^{2}}$$

where c is the humidity (the ratio of water mass per unit area to the mass of the dry material), $D_{z}$ is the material diffusion coefficient in the thickness direction, and t is time.

The change in moisture absorption $M_{r}$ of a single-layer composite plate can be represented by the change in weight, as shown in Equation (2), and it is also a function of t.

(2) $$M_{r}=M_{r}\left(t\right)=\frac{m\left(t\right)-m_{\mathrm{dry}}}{m_{\mathrm{dry}}}\times 100$$

where $m\left(t\right)$ is the mass of the single-layer plate after absorbing water, and $m_{\mathrm{dry}}$ is the mass of the single-layer plate when dry.

The mass of the single-layer plate when saturated with moisture is denoted as $m_{m}$, then the mass of the single-layer plate at any time $m\left(t\right)$ can be represented by Equation (3), and the moisture absorption rate can be represented by Equation (5).

The mass of the single-layer plate when saturated with moisture is denoted as $m_{m}$. The mass of the single-layer plate at any time $m\left(t\right)$ can be represented by Equation (3). Based on the definition provided in Equation (2), subtracting $m_{\mathrm{dry}}$ from both sides of the equation and dividing the result by $m_{\mathrm{dry}}$ leads to Equation (4). Consequently, the moisture absorption rate $M_{r}$ can be represented by Equation (5).

(3) $$m\left(t\right)=m_{\mathrm{water}}+m_{0}=G\left(m_{m}-m_{0}\right)+m_{0}$$

(4) $$\frac{m\left(t\right)-m_{\mathrm{dry}}}{m_{\mathrm{dry}}}=\frac{G\left(\left(m_{m}-m_{\mathrm{dry}}\right)-\left(m_{0}-m_{\mathrm{dry}}\right)\right)}{m_{\mathrm{dry}}}+\frac{m_{0}-m_{\mathrm{dry}}}{m_{\mathrm{dry}}}$$

(5) $$M_{r}=G\left(M_{m}-M_{0}\right)+M_{0}$$

where $m_{\mathrm{water}}$ is the increase in mass of the single-layer plate due to moisture absorption, and $m_{0}$ is the mass of the single-layer plate at the initial time. If the single-layer plate is initially in a dry state, $m_{0}=m_{\mathrm{dry}}$; if the single-layer plate contains a certain amount of moisture at the initial time, $m_{0}>m_{\mathrm{dry}}$. G is the saturation degree, reaching 1 when saturated with moisture. The expression for it can be obtained by solving Fick’s second law, as shown in Equation (10). $M_{m}$ is the moisture absorption rate of the single-layer plate in the saturated state, and $M_{0}$ is the moisture absorption rate of the single-layer plate at the initial time. When the initial time is in a dry state, $M_{0}=0$.

**Figure 1:**
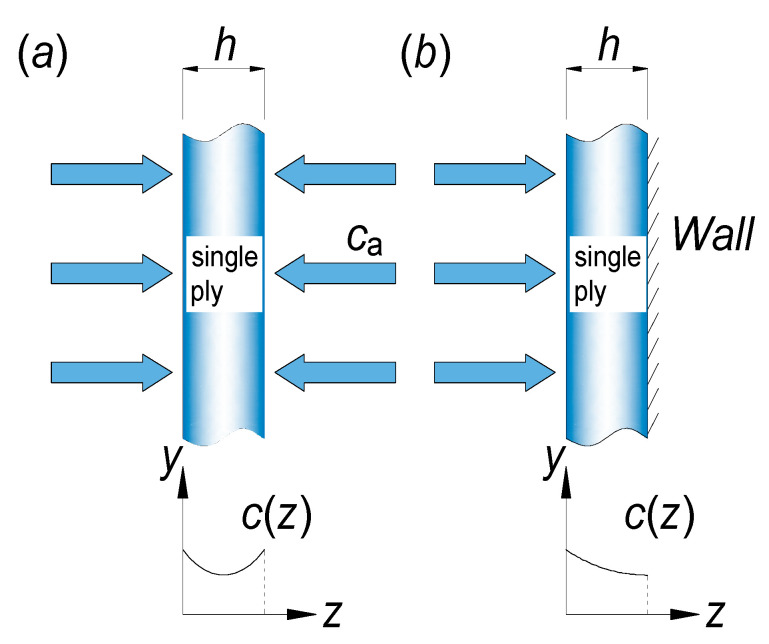
Variation in moisture content within a composite plate. (**a**) When both sides are exposed to the environment, the equivalent thickness s=h; (**b**) when only a single side is exposed, s=2h.

Assuming an infinite plate, the humidity of the composite single-layer plate is assumed to vary only in the thickness direction z. At the initial time, the humidity $c_{0}$ inside the single-layer plate is uniformly distributed in the thickness direction z. Subsequently, both sides of the single-layer plate are exposed to an environment with humidity $c_{a}$, as illustrated in Figure 1a. Substituting the above boundary conditions into the differential Equation (1) yields the humidity distribution inside the single-layer plate.

(6) $$\left\{\begin{array}{c} \frac{\partial c}{\partial t}=D_{z}\frac{\partial^{2}c}{\partial z^{2}}\\ c=c_{0},\;0<z<h,\;t=0\\ c=c_{a},\;z=0;z=h,\;t>0 \end{array}\right.$$

(7) $$\frac{c-c_{0}}{c_{m}-c_{0}}=1-\frac{4}{\pi }\sum_{n=0}^{\infty }\frac{1}{\left(2n+1\right)}\sin\frac{\left(2n+1\right)\pi z}{h}\exp\left[-{\left(2n+1\right)}^{2}\pi^{2}\frac{D_{z}t}{s^{2}}\right]$$

where s is the equivalent thickness. When both sides of the single-layer plate are in a hygrothermal environment, as shown in Figure 1a, the equivalent thickness is equal to the actual thickness, i.e., s=h. If only one side of the single-layer plate is exposed to a hygrothermal environment, water molecules diffuse from the exposed side through to the opposite side. This diffusion process is equivalent to a scenario where both sides are exposed, with water molecules diffusing towards the mid-plane from both sides. Therefore, the equivalent thickness, accounting for this diffusion behavior, is twice the actual thickness, i.e., s=2h. If the single-layer plate is in a dry state at the initial time, then $c_{0}=0$.

The mass of moisture per unit area within a single-layer plate can be obtained by integrating the humidity along the thickness direction, as expressed in Equation (8).

(8) $$m_{\mathrm{water}}=\int_{0}^{h}cdz$$

(9) $$\left\{\begin{array}{l} m\left(t\right)-m_{0}=\int_{0}^{h}\left(c-c_{0}\right)dz\\ m_{m}-m_{0}=\int_{0}^{h}\left(c_{m}-c_{0}\right)dz \end{array}\right.$$

Based on the definition of saturation G in Equation (3), it can be expressed as follows:

(10) $$G=\frac{m\left(t\right)-m_{0}}{m_{m}-m_{0}}=1-\frac{8}{\pi^{2}}\sum_{n=0}^{\infty }\frac{1}{{\left(2n+1\right)}^{2}}\exp\left[-{\left(2n+1\right)}^{2}\pi^{2}\frac{D_{z}t}{h^{2}}\right]$$

The infinite series summation in Equation (10) can be obtained using the function fitting method [23], as shown in Equation (11).

(11) $$G=1-\exp\left[-7.3{\left(\frac{D_{z}t}{h^{2}}\right)}^{0.75}\right]$$

The relationship between $M_{r}$ of the single-layer plate and t can be expressed as follows:

(12) $$M_{r}=\left[1-\exp\left[-7.3{\left(\frac{D_{z}t}{h^{2}}\right)}^{0.75}\right]\right]\left(M_{m}-M_{0}\right)+M_{0}$$

The above derivation is based on the assumption of an infinitely large plate, while the actual sample in the experiment is elongated, and moisture molecules will diffuse into the interior of the single-layer plate on all surfaces of the sample [24]. Therefore, it is necessary to make corrections to $D_{z}$ and a more accurate diffusion coefficient can also be determined through experimental measurements.

(13) $$D_{z}^{\prime }=D_{z}\left(1+\frac{h}{l}\sqrt{\frac{D_{x}}{D_{z}}}+\frac{h}{n}\sqrt{\frac{D_{y}}{D_{z}}}\right)$$

where l is the length of the single-layer plate sample; n is the width of the single-layer plate sample; h is the thickness of the single-layer plate sample; $D_{x}$ is the diffusion coefficient in the length direction of the single-layer plate; $D_{y}$ is the diffusion coefficient in the width direction of the single-layer plate, which can be given by the heat and mass transfer model based on the subregion method [23,25].

(14) $$D_{1}=v_{f}D_{f}+v_{m}D_{m}$$

(15) $$D_{2}=D_{3}=\left(\frac{\left[\pi -\frac{4}{\sqrt{1-\left(A^{2}v_{f}/\pi \right)}}\tan^{-1}\frac{\sqrt{1-\left(A^{2}v_{f}/\pi \right)}}{1+\sqrt{A^{2}v_{f}/\pi }}\right]}{A}+\left(1-2\sqrt{v_{f}/\pi }\right)\right)D_{m}$$

where $A=2(D_{m}/D_{f}$ − 1); $D_{m}$ is the moisture diffusion coefficient of the matrix; $D_{f}$ is the moisture diffusion coefficient of the carbon fiber; $v_{f}$ is the volume fraction of the fiber in the single-layer plate; $v_{m}$ is the volume fraction of the matrix in the single-layer plate; subscripts 1, 2, and 3 represent the coordinates of the fiber in the positive axis coordinate system, while subscripts x, y, and z represent the coordinates of the rectangular coordinate system of the single-layer plate. When the single-layer plate is laid at 0°, the xyz coordinate system coincides with the 123 coordinate system. When the single-layer plate is laid at other angles, the moisture diffusion coefficient in the xyz coordinate system can be calculated through the transformation formula.

### 2.2. Model for Mechanical Performance Under Hygrothermal Aging

The impact of hygrothermal environment on the mechanical behavior of single-layer plates can be summarized into two main aspects: (1) Firstly, there is a change in stress–strain distribution, mainly caused by the swelling and plasticization of the matrix, which are reversible reactions [26,27]; (2) secondly, there is performance degradation, mainly caused by the hydrolysis and post-curing of the matrix, which are irreversible reactions [11]. This study focuses on CFRP tensile specimens, and the stress–strain can be determined by analyzing the plane stress state. Meanwhile, the degradation of composite material performance can be described using the model established in this study.

When developing the mathematical model for the hygrothermal performance of single-layer plates, it is assumed that the mechanical and hygrothermal effects are linear and can be processed separately and then superimposed. The plane stress state of the single-layer plate in a hygrothermal environment is as follows:

(16) $$\sigma =\bar{Q}\left(\varepsilon -\alpha \Delta T-\beta c\right)$$

where $\bar{Q}$ is the bending stiffness matrix of the single-layer plate; α is the coefficient of thermal expansion (CTE) of the single-layer plate; $\Delta T=T-T_{0}$ is the temperature change, where T is the final temperature and $T_{0}$ is the initial temperature; β is the coefficient of hygroscopic expansion (CHE) of the single-layer plate.

The last two terms in the parentheses in Equation (16) represent the strain increment directly caused by temperature and humidity changes. It is important to note that the bending stiffness matrix, CTE, and CHE of the single-layer plate will also change with temperature and humidity. In this study, an empirical formula is used to establish the relationship between the glass transition temperature $T_{g}$ and the matrix properties, which are then incorporated into the micro-mechanical model to obtain the stiffness matrix in a hygrothermal environment.

Based on experimental observations, it was found that the stiffness of the matrix gradually decreases as the temperature rises until it reaches the glass transition temperature $T_{\mathrm{gw}}$, after which it rapidly decreases [1]. Building on this observation, Chamis and Sinclair described the process of hygrothermal aging using Equation (17) [28].

(17) $$F_{m}=\frac{P}{P_{0}}={\left(\frac{T_{\mathrm{gw}}-T}{T_{g0}-T_{0}}\right)}^{\frac{1}{2}}$$

where $F_{m}$ is the retention rate of the matrix’s mechanical properties; $P_{0}$ is the stiffness of the matrix in the ambient temperature; P is the stiffness of the matrix after hygrothermal aging; $T_{g0}$ is the glass transition temperature of the matrix under dry and ambient conditions, in °F; $T_{0}$ is the ambient temperature, in °F; T is the temperature of the hygrothermal environment, in °F; $T_{\mathrm{gw}}$ is the glass transition temperature of the matrix in the hygrothermal environment, in °F. $T_{\mathrm{gw}}$ decreases with increasing moisture absorption rate and can be described by Equation (18).

(18) $$T_{\mathrm{gw}}=\left(0.005M_{r}^{2}-0.01M_{r}+1\right)T_{g0}$$

Equation (18) is derived from experiments on the epoxy resin matrix and should be validated using experimental data when applied to other matrices. According to the micromechanics model, the modulus of a single-layer laminate after hygrothermal aging in the fiber axial direction $E_{1}$ can be calculated using a mixing rule.

(19) $$E_{1}=E_{f1}v_{f}+F_{m}E_{m}v_{m}=E_{f1}v_{f}+{\left(\frac{\left(0.005M_{r}^{2}-0.01M_{r}+1\right)T_{\mathrm{go}}-T}{T_{\mathrm{go}}-T_{o}}\right)}^{\frac{1}{2}}E_{m}v_{m}$$

where $v_{f}$ and $v_{m}$ represent the volume fractions of the fiber and matrix in the single-layer laminate, respectively; $E_{f1}$ is the tensile modulus of the fiber in the direction 1; $E_{m}$ is the tensile modulus of the matrix. Since the mechanical properties of carbon fiber are significantly less affected by the hygrothermal environment compared to the matrix, the degradation of the mechanical properties of the carbon fiber is neglected. The degradation of the mechanical properties of the matrix is only considered in the influence on the mechanical properties of the composite material.

The directional 2 (transverse) modulus $E_{2}$ and the 1–2 in-plane shear modulus $G_{12}$ of the single-layer laminate can be calculated using the subregion method, Spencer’s formula, the Halpin–Tsai formula, or the Tsai–Hahn formula. The results obtained from these methods are in good agreement with experimental results. However, Spencer’s formula requires solving implicit functions, which can be operationally challenging. The Halpin–Tsai formula and the Tsai–Hahn formula depend on curve fitting parameters and stress partitioning parameters, and they have a semi-empirical nature. In this study, the theoretically derived subregion method is adopted, and the transverse modulus $E_{2}$ of the single-layer laminate after hygrothermal aging is as follows:

(20) $$E_{2}=F_{m}E_{m}\left[\left(1-\sqrt{v_{f}}\right)+\frac{\sqrt{v_{f}}}{1-\sqrt{v_{f}}\left(1-F_{m}E_{m}/E_{f2}\right)}\right]=E_{3}$$

where $E_{3}$ is the directional 3 tensile modulus of the single-layer laminate, and $E_{f2}$ is the tensile modulus in the fiber direction 2, with the meanings of other symbols being the same as mentioned earlier.

The shear modulus $G_{12}$ and $G_{23}$ of the single-layer laminate after hygrothermal aging is represented by Equations (21) and (22).

(21) $$G_{12}=F_{m}G_{m}\left[\left(1-\sqrt{v_{f}}\right)+\frac{\sqrt{v_{f}}}{1-\sqrt{v_{f}}\left(1-F_{m}G_{m}/G_{f12}\right)}\right]=G_{13}$$

(22) $$G_{23}=F_{m}G_{m}\left[\left(1-\sqrt{v_{f}}\right)+\frac{\sqrt{v_{f}}}{1-\sqrt{v_{f}}\left(1-F_{m}G_{m}/G_{f23}\right)}\right]$$

where $G_{12}$ is the shear modulus of the single-layer laminate in the 1–2 plane, $G_{13}$ is the shear modulus of the single-layer laminate in the 1–3 plane, $G_{23}$ is the shear modulus of the single-layer laminate in the 2–3 plane, $G_{f12}$ is the shear modulus of the fiber in the 1–2 plane, and $G_{23}$ is the shear modulus of the fiber in the 2–3 plane.

Due to the significantly lower tensile modulus of the matrix compared to the carbon fiber, it can be inferred from Equation (19) that the longitudinal modulus of the single-layer laminate is predominantly influenced by the properties of the carbon fiber. Additionally, Equations (20) and (21) indicate that the transverse modulus and shear modulus of the single-layer laminate are primarily determined by the properties of the matrix. Moreover, as the mechanical performance of the matrix is significantly more susceptible to the effects of the hygrothermal environment than the carbon fiber, the transverse modulus and shear modulus of the single-layer laminate are considerably more influenced by the hygrothermal environment than the longitudinal modulus.

The principal Poisson’s ratio $v_{12}$ of the single-layer laminate can be determined using the mixture rule, as demonstrated in Equation (23).

(23) $$\upsilon_{21}=\upsilon_{12}E_{2}/E_{1}=\left(\upsilon_{f12}v_{f}+\upsilon_{m}v_{m}\right)E_{2}/E_{1}$$

where $v_{f12}$ is the principal Poisson’s ratio of the fiber, and $v_{m}$ is the Poisson’s ratio of the matrix.

According to the Maxwell reciprocity theorem, the secondary Poisson’s ratio $v_{21}$ can be expressed as follows:

(24) $$\upsilon_{21}=\upsilon_{12}E_{2}/E_{1}=\left(\upsilon_{f12}v_{f}+\upsilon_{m}v_{m}\right)E_{2}/E_{1}$$

It can be observed from Equation (24) that the 21-direction Poisson’s ratio is related to the tensile modulus in the corresponding direction of the single-layer laminate, and the tensile modulus of the single-layer laminate is closely linked to the tensile modulus of the matrix. Therefore, the 21-direction Poisson’s ratio will also undergo a certain degree of change in a hygrothermal environment.

## 3. Experimental Section

The hygrothermal aging model for mechanical performance established in the previous section was experimentally validated. Firstly, the moisture absorption and diffusion model was verified through seawater immersion experiments. Finally, mechanical performance tests were conducted on the specimens after seawater immersion, confirming the capability of the hygrothermal aging model to predict the mechanical properties of composite materials.

### 3.1. Experimental Materials

The CFRP composite material utilized in the experiments was fabricated using the wet winding process. A flat plate served as the mold for continuous winding of four layers. The density of the CFRP was measured to be 1.56 ± 0.06 $g/{\mathrm{cm}}^{3}$ using the drainage method, and the mass fraction of carbon fiber was determined to be 69.8 ± 1.12% using the combustion method according to the ASTM D3171-22 [29], which is approximately equivalent to a volume fraction of 60.49 ± 3.33%. The carbon fiber used was domestically produced T700 carbon fiber, and the matrix was a thermosetting epoxy resin, with performance parameters as shown in Table 1 and Table 2.

### 3.2. Composite Material Seawater Immersion Experiment

**Figure 2:**
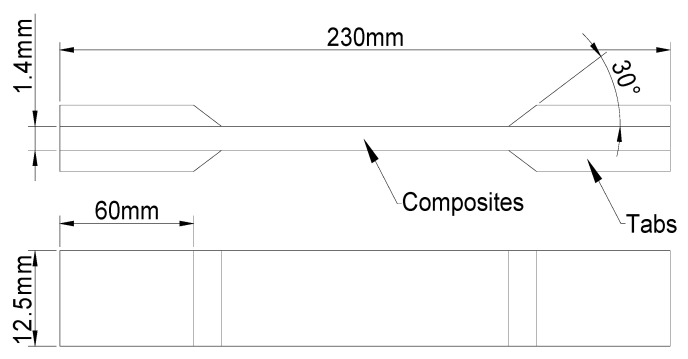
Schematic diagram of specimen size.

The dimensions of the CFRP specimen were based on the GB/T 3354-2014 [30], as illustrated in Figure 2. The specimen was a unidirectional tape, with the fiber axis angle at 0° to the length direction of the specimen, and the specimen size was 230 × 12.5 × 1.4 mm^3^. To prevent gripping damage to the specimens, short-cut glass fiber tabs were employed at the gripping positions and adhered using a thermosetting adhesive. The dimensions of the tabs, as illustrated in Figure 2, were designed to ensure the minimum required bonding length while maximizing the gripping surface area.

**Figure 3:**
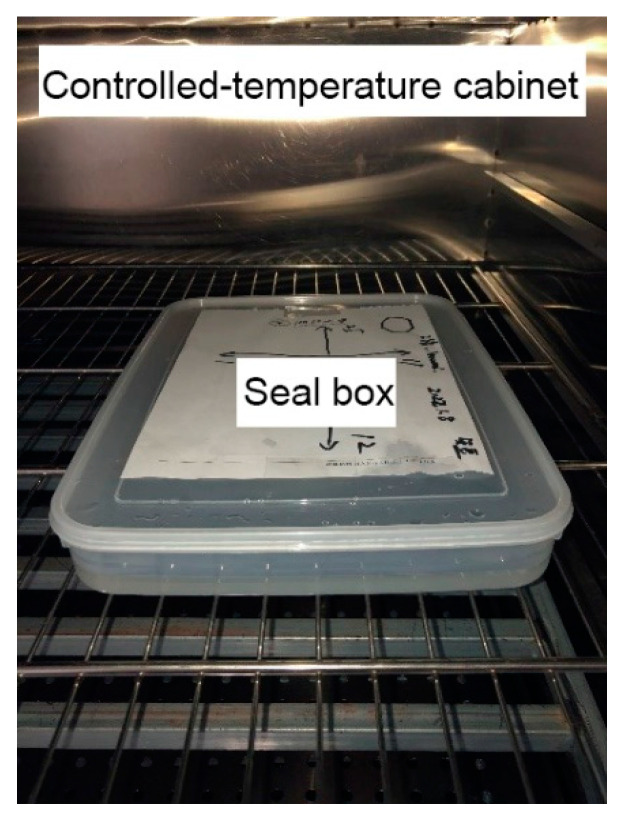
Seawater immersion test for composite materials.

**Figure 4:**
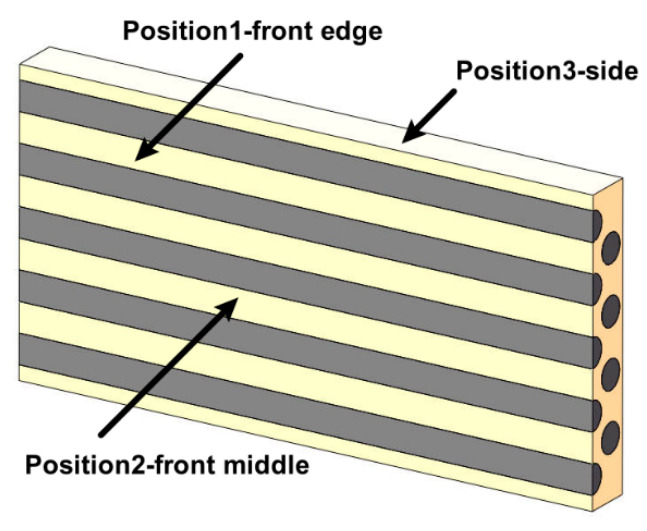
Schematic of sampling observation position.

Following the ASTM D5229/D5229M-20 [31], the weighing method was employed to evaluate the hygroscopic properties of composite materials. Initially, the composite material specimens were placed in a constant temperature box at 40 ± 1 °C (101-2A/B, Qiuzuo Scientific Instrument, Shanghai, China) to remove the initial moisture until the specimens reached a constant weight. Subsequently, the specimens were immersed in a container filled with seawater at room temperature (25 ± 1 °C) for moisture absorption, as depicted in Figure 3. The seawater was collected from the coastal waters off Zhoushan, Zhejiang Province, China. After evaporating the water, the coarse salt content was measured to be 33.77 ± 8.61 g/L by weight. Each group consisted of 5 replicated specimens, and the specimens were taken out for weighing at 1, 2, 5, 8, 10, 20, 30, 60, 100, and 658 days, using an electronic balance with an accuracy of ±1 mg. Before weighing, filter paper was used to absorb the moisture on the specimen surface. The moisture absorption rate inside the composite material at each time point was calculated according to Equation (2). The surface morphology of the composite material was periodically observed under an optical microscope for changes over a period of 60 days, with the sampling observation positions shown in Figure 4. The rationale for selecting the front edge and side surfaces as an observation point is that it is typically where stress concentration and corrosion initiation occur. Edge regions are also more susceptible to environmental factors, thus better reflecting the corrosion behavior under actual usage conditions [32].The midpoint of the front surface is generally considered the corrosion effective zone, where the corrosion environment is relatively uniform. This area can more accurately reflect the overall average corrosion rate and changes in physical properties of the material. Observations here provide insights into the general corrosion performance.

Therefore, these three points were selected as observation points to comprehensively evaluate the corrosion behavior and physical property changes of the composite material under study.

### 3.3. Mechanical Performance Testing

**Figure 5:**
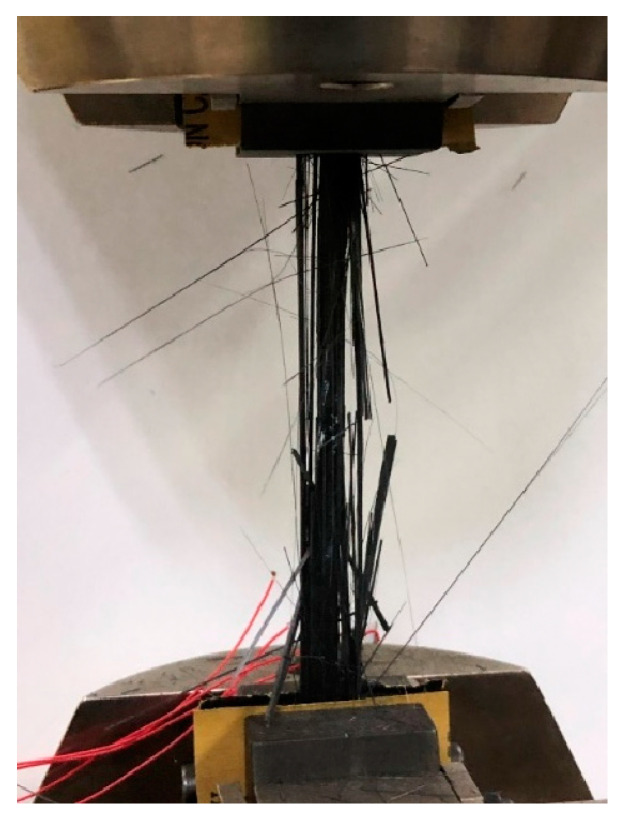
Mechanical properties testing of composites.

Following the GB/T 3354-2014 [30], the tensile mechanical properties are tested using a tensile testing machine (PLW-100KN, Shenli Testing Machine, Shanghai, China) equipped with hydraulic-driven wedge grips. The sample is loaded continuously at a speed of 2 mm/min until failure, as illustrated in Figure 5. Data are recorded and used to calculate the tensile modulus and tensile strength of the specimen. For the tensile chord modulus calculation, the longitudinal strain range used was from 0.001 to 0.003.

## 4. Results and Discussion

**Figure 6:**
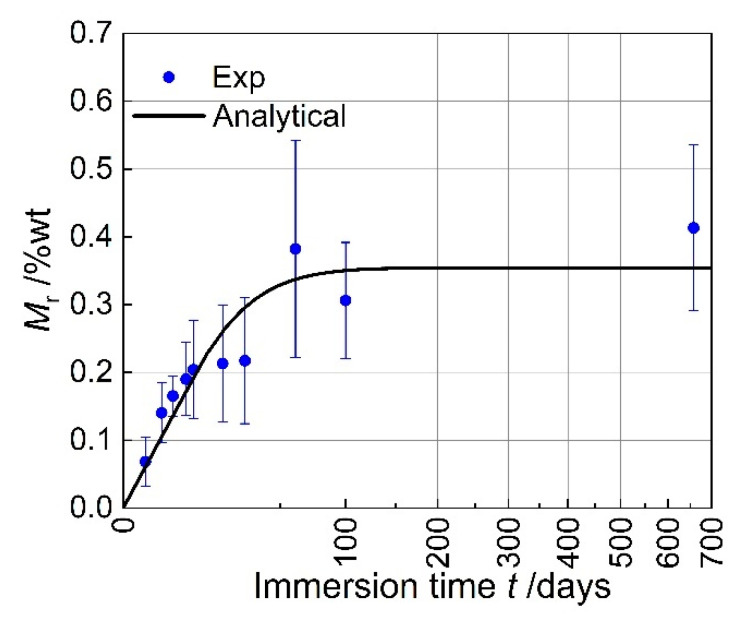
Results and fitted curves of seawater immersion test at room temperature.

The model established in the preceding text, as shown in Equation (12), describes the variation in the moisture absorption rate $M_{r}$ with immersion time t. Substituting the experimental data into Equation (12) for fitting yields the experimental results and fitting curve for moisture absorption in composite materials, as illustrated in Figure 6. In this figure, the horizontal axis represents the square root transformation of time.

From Figure 6, it can be observed that the moisture absorption rate of the composite material initially increases linearly with the square root of time, and then stabilizes, which is consistent with Fick’s second law of diffusion. The parameters of the model are given in Equation (25), where the saturation moisture absorption rate $M_{m}$ is determined to be 0.3544%. According to one study, the saturation moisture absorption rate $M_{m,Epoxy}$ of epoxy resin is in the range of 0.7% to 1.5% [33]. Given that the mass fraction of carbon fiber $w_{f}$ is approximately 69.8%, the saturation moisture absorption rate of the composite material $M_{m}=M_{m,Epoxy}=0.211\sim 0.453\%$, which is consistent with the experimental results.

(25) $$M_{r}=0.3544\left[1-\exp\left[-7.3{\left(\frac{0.009573t}{1.361^{2}}\right)}^{0.75}\right]\right]$$

where t is the immersion time in days, and $M_{r}$ is the moisture absorption rate in percentage.

**Figure 7:**
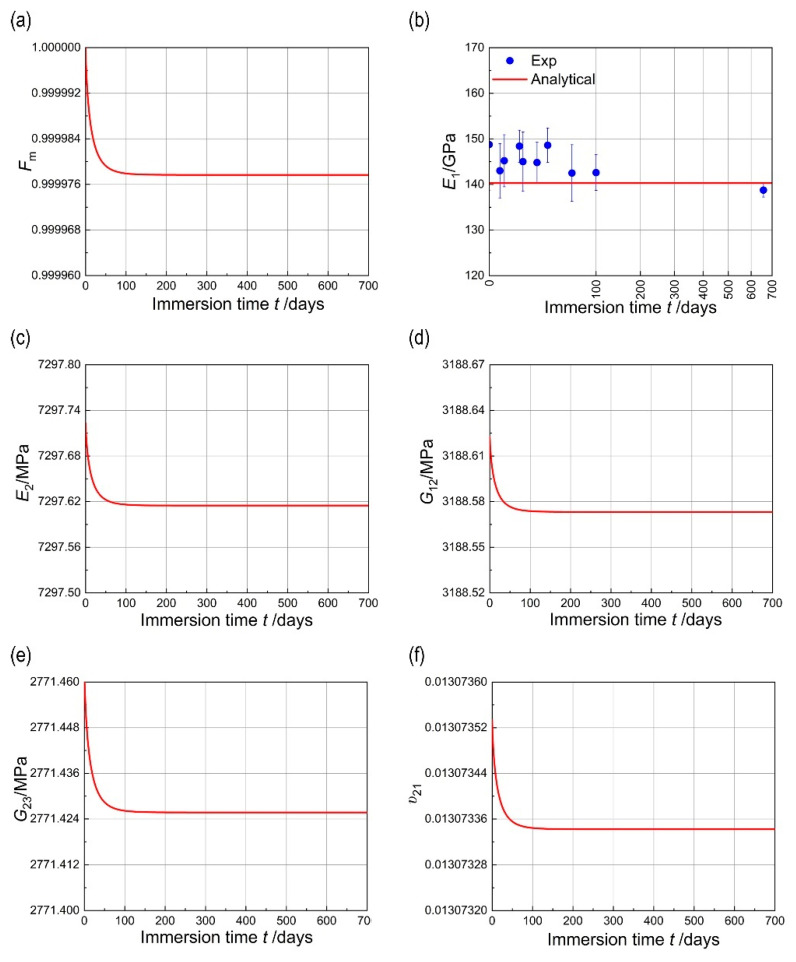
(**a**) Variation in matrix property retention with immersion time; (**b**) variation of $E_{1}$ with immersion time; (**c**) variation of $E_{2}$ with immersion time; (**d**) variation of $G_{12}$ with immersion time; (**e**) variation of $G_{23}$ with immersion time; (**f**) variation of $v_{21}$ with immersion time.

As shown in Figure 7, the variation in the matrix performance retention rate with immersion time is calculated using Equation (17). It is evident from the figure that the matrix performance exhibits a decreasing trend with increasing immersion time. After reaching a certain time threshold, the matrix reaches the moisture saturation state, and the mechanical properties of the composite material tend to stabilize.

From the perspective of the retention rate, it is evident that the hygroscopic effect under room temperature within 658 days has a negligible impact on the mechanical properties of the composite material. This is further evidenced by the experimental results of the mechanical properties of the composite material. As depicted in Figure 7b, the trend of the tensile modulus $E_{1}$ of the single-layer plate with immersion time closely aligns with the analytical solution. During the immersion of the composite material in seawater, the carbon fiber itself exhibits a much lower moisture absorption rate compared to the resin matrix. Consequently, the impact of seawater immersion on the axial modulus of the carbon fiber is smaller than that of the resin matrix. As the axial modulus of $E_{1}$ is primarily determined by the carbon fiber, the decreasing trend with immersion time indicates a relatively minor degree of degradation.

After 700 days of immersion, the model predicts that $E_{1}$, $E_{2}$, $G_{12}$, $G_{23}$, and $v_{21}$ decrease by 2.14 × 10^−5^%, 1.49 × 10^−3^%, 1.55 × 10^−3^%, 1.37 × 10^−3^%, and 1.47 × 10^−3^%, respectively (Figure 7). This is attributed to the higher degradation degree of the matrix modulus compared to the carbon fiber after immersion in seawater. Additionally, the degradation of the composite material’s transverse modulus and shear modulus is primarily influenced by the matrix modulus, resulting in greater degradation of $E_{2}$, $G_{12}$, and $G_{23}$ compared to $E_{1}$.

**Figure 8:**
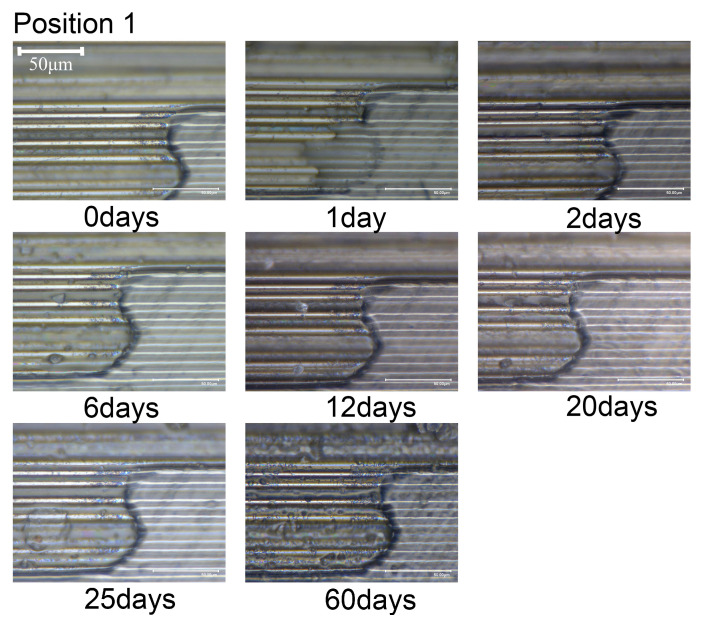
Variation in morphology with immersion time at position 1.

**Figure 9:**
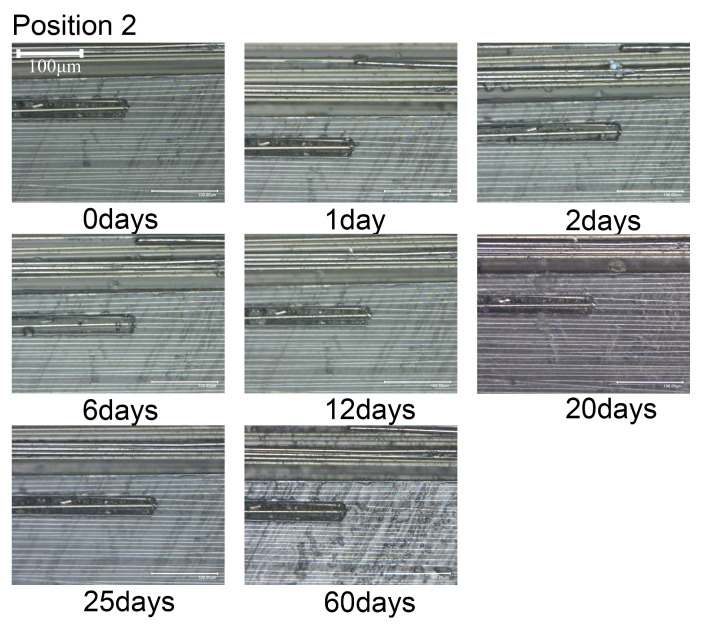
Variation in morphology with immersion time at position 2.

**Figure 10:**
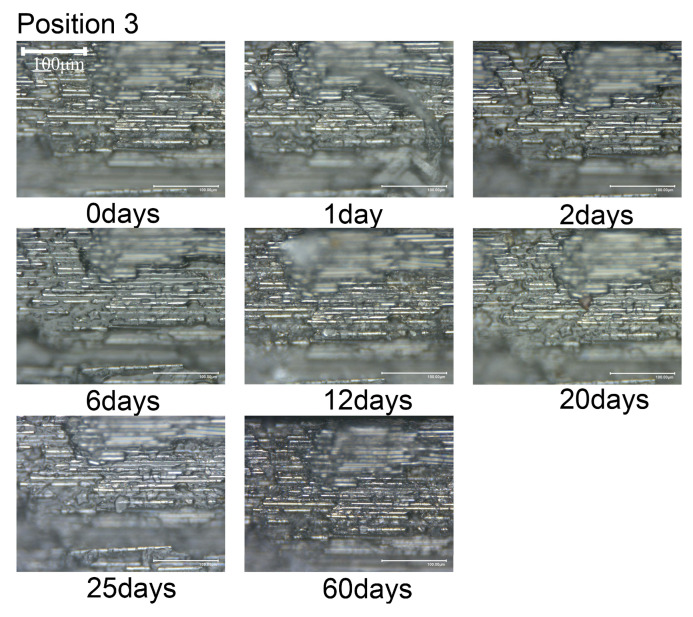
Variation in morphology with immersion time at position 3.

The microstructural changes over time are illustrated in Figure 8, Figure 9 and Figure 10. When the composite material is immersed in seawater at room temperature for 60 days, transparent crystals precipitate at observing locations 1 and 2, without any significant change in the surface morphology. Hence, it can be inferred that short-term seawater immersion (≤60 days) at room temperature will not result in surface cracks or defects (≥10 μm) in the composite material.

**Figure 11:**
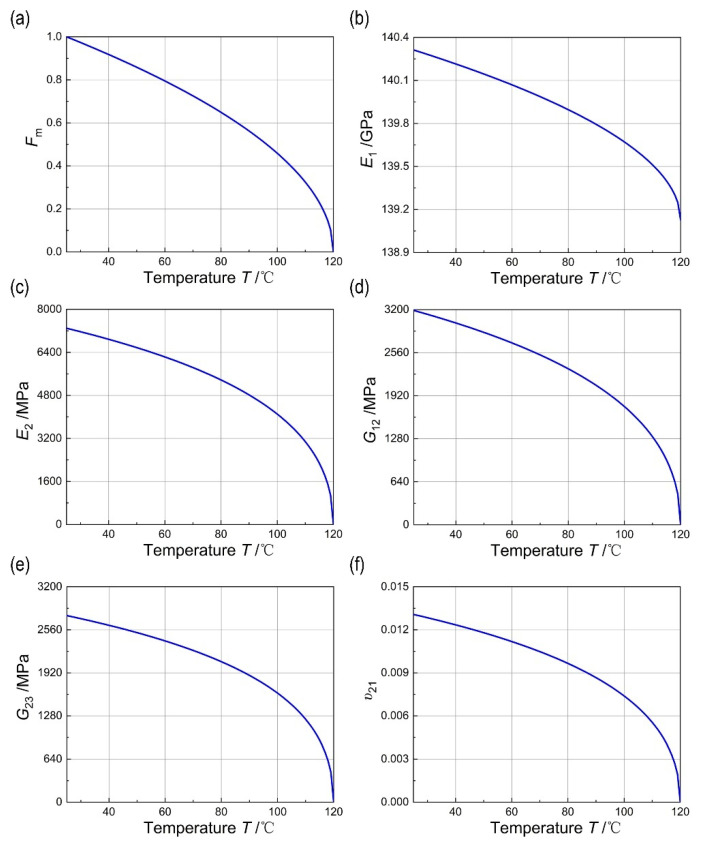
(**a**) Variation in matrix property retention with temperature; (**b**) variation of $E_{1}$ with temperature; (**c**) variation of $E_{2}$ with temperature; (**d**) variation of $G_{12}$ with temperature; (**e**) variation of $G_{23}$ with temperature; (**f**) variation of $v_{21}$ with temperature.

Furthermore, Equation (17) indicates that the mechanical properties of the composite material will change with temperature. Figure 11a illustrates the variation in the matrix performance retention rate with temperature. It is evident from the figure that the matrix performance decreases as the temperature rises, and when the temperature reaches the glass transition temperature $T_{g}$ of the matrix, the matrix performance drops to 0, rendering the matrix incapable of supporting the carbon fibers and bearing the load. The rate of decrease in the matrix performance is higher as the working temperature T approaches $T_{g}$.

As shown in Figure 11, the tensile modulus along the fiber axis of the composite material shows little change with the increase in temperature, whereas the tensile modulus $E_{2}$ in the fiber cross-sectional direction decreases to 0 as the temperature rises. Similarly, the shear modulus $G_{12}$ and $G_{23}$ also exhibits a decreasing trend with the temperature rise, eventually reaching 0. This is attributed to the fact that the transverse modulus and shear modulus of the composite material are primarily determined by the matrix. When the matrix loses its load-bearing capacity, both the transverse modulus and shear modulus decrease to 0. After heating to 120 °C, the hygrothermal aging model in this paper predicts a decrease of $E_{1}$, $E_{2}$, $G_{12}$, $G_{23}$, and $v_{21}$ in 0.84%, 100%, 100%, 100%, and 100%, respectively.

## 5. Conclusions

This study thoroughly examines both the hygrothermal aging behavior of composite materials in seawater environments and the effects on their mechanical properties. An aging model is developed to forecast changes in performance across various conditions. By integrating empirical data with theoretical analysis, the suitability of Fick’s second law for characterizing the moisture absorption process is confirmed. Additionally, this research elucidates the intricate interplay among temperature, moisture absorption rate, and the mechanical properties of composites. The insights gained offer a scientific foundation for assessing the long-term stability of composite materials in marine settings. In light of these findings, potential avenues for future research are outlined, intended to stimulate further innovation and exploration in the field.

This study of the evolution of the moisture absorption rate in composite materials is described according to Fick’s second law. It further explores the relationship between the mechanical properties of the matrix component, the moisture absorption rate, and temperature. Combined with a micromechanical model, an aging model is established to predict the changes in the tensile modulus, shear modulus, and Poisson’s ratio of the composite materials with variations in moisture absorption rate and temperature.

In a seawater environment, the T700 carbon fiber/epoxy resin-based composite single-layer plate has a saturated moisture absorption rate of 0.35%, reaching the saturation state at around 700 days. Surface morphology observation indicates that short-term seawater immersion at room temperature (≤60 days) does not lead to the production of cracks or defects (≥10 μm) on the composite material’s surface.

The model calculations and experimental results indicate that the modulus of the composite material decreases with the increase in moisture absorption rate. The longitudinal tensile modulus, predominantly composed of carbon fibers, decreases by an order of 10^−7^ magnitude, while the transverse tensile modulus, shear modulus, and secondary Poisson’s ratio, predominantly composed of the matrix, also decrease by an order of 10^−5^ magnitude.

The model calculations demonstrate that the modulus of the composite material decreases with the rise in temperature, and the closer it gets to the glass transition temperature of the matrix, the faster the modulus decreases. During the process from room temperature to the glass transition temperature of the matrix, the decrease in the longitudinal tensile modulus, predominantly composed of carbon fibers, is 0.84%, and the decrease in the transverse tensile modulus, shear modulus, and secondary Poisson’s ratio, predominantly composed of the matrix, is 100%, causing the epoxy resin matrix to lose its load-bearing capacity. Therefore, when composite materials are applied in high-temperature environments, it is essential to avoid exceeding $T_{g}$ of the matrix and to orient the primary load-bearing direction along the length of the composite material fibers.

To further refine the model, the next steps should include conducting high-temperature tests and evaluating the shear modulus. Given the significant influence of temperature on composite material properties, especially as temperatures approach $T_{g}$ of the matrix, enhancing the stability of these materials under high-temperature and high-humidity conditions through improvements in manufacturing processes or the incorporation of functional additives becomes critical.

Future research should focus on investigating the performance changes of composite materials under various hygrothermal cycling conditions and assessing the long-term effects of hygrothermal aging on material properties. Such studies will provide crucial insights into the challenges that materials might face in extreme environments encountered during real-world applications.

## Figures and Tables

**Table 1:** Material properties of the T700.

Parameter	Sample	Unit	T700
Density	ρ_f_	g∙cm^−3^	1.8
Fiber diameter	d_f_	μm	7.62
1-direction tensile modulus	E_f1_	GPa	230
2-direction tensile modulus	E_f2_	GPa	18
12-direction shear modulus	G_f12_	GPa	8.7
23-direction shear modulus	G_f23_	GPa	5.8
12-direction Poisson’s ratio	υ_f12_		0.2
23-direction Poisson’s ratio	υ_f23_		0.49

**Table 2:** Material properties of the epoxy.

Parameter	Sample	Unit	AF-4206A/B
Density	ρ_f_	g∙cm^−3^	1.2
Tensile modulus	E_m_	GPa	≥3.0
Shear modulus	G_m_	GPa	≥1.25
Poisson’s ratio	υ_m_		0.33
Heat conductivity	K_m_	W∙mm^−1^∙°C^−1^	1.8034 × 10^−4^
Specific heat capacity at constant volume	C_m,v_	J∙kg^−1^∙°C^−1^	1046.7
Coefficient of thermal expansion	α_m_	mm∙mm^−1^∙°C^−1^	6.48 × 10^−5^
Coefficient of hydroscopic expansion	β_m_	mm∙mm^−1^∙%^−1^	0.33
Diffusion coefficient	D_m_	mm^2^∙s^−1^	0.25 × 10^−6^
Glass transition temperature	T_g_	°C	≥120

## Data Availability

The original contributions presented in this study are included in the article. Further inquiries can be directed to the corresponding author.
